# Systematization of Nursing Care in undergraduate training: the perspective of Complex Thinking^1^

**DOI:** 10.1590/0104-1169.0096.2525

**Published:** 2015

**Authors:** Josilaine Porfírio da Silva, Mara Lucia Garanhani, Aida Maris Peres

**Affiliations:** 2 MSc, RN, Hospital Zona Sul de Londrina, Londrina, PR, Brazil; 3 PhD, Professor, Departamento de Enfermagem, Universidade Estadual de Londrina, Londrina, PR, Brazil; 4 PhD, Professor, Departamento de Enfermagem, Universidade Federal do Paraná, Curitiba, PR, Brazil

**Keywords:** Education, Nursing, Nursing Education Research, Students, Nursing, Nursing Process, Nursing, Curriculum

## Abstract

**AIM:**

to evaluate the clinical applicability of outcomes, according to the Nursing Outcomes Classification (NOC) in the evolution of orthopedic patients with Impaired Physical Mobility

**METHOD:**

longitudinal study conducted in 2012 in a university hospital, with 21 patients undergoing Total Hip Arthroplasty, evaluated daily by pairs of trained data collectors. Data were collected using an instrument containing five Nursing Outcomes, 16 clinical indicators and a five point Likert scale, and statistically analyzed.

**RESULTS:**

The outcomes Body Positioning: self-initiated, Mobility, Knowledge: prescribed activity, and Fall Prevention Behavior presented significant increases in mean scores when comparing the first and final evaluations (p<0.001) and (p=0.035).

**CONCLUSION:**

the use of the NOC outcomes makes it possible to demonstrate the clinical progression of orthopedic patients with Impaired Physical Mobility, as well as its applicability in this context.

## Introduction

The Systematization of Nursing Care (SAE) is regulated, in Brazil, as a method which organizes professional work, making possible the implementation of the nursing process (NP), a methodological instrument which guides the professional nursing care, and which is organized in five interrelated stages: data collection, nursing diagnosis, planning, implementation and nursing evaluation^(^ 1 ^)^.

The use of a scientific instrument ensures the professional the qualification of the management of the care, and the planning of her activities, as well as serving as a guide for her actions^(^ 2 ^)^. In this perspective, the use of SAE affords not only individualized care, but also allows greater visibility to her actions^(^ 3 ^-^ 4 ^)^.

This issue has been the object of study in nursing schools, due to the recognition of its importance in the training and work of the nurse, which leads to the question of how SAE has been undertaken in the undergraduate courses, as this is when the student has contact with the basics of her profession and begins the formation of her professional attitude.

In order to better understand the training of the students for undertaking SAE, it was decided to study one undergraduate course which uses a different pedagogical proposal, the integrated curriculum. In this, the content is integrated and the curricular matrix is structured in interdisciplinary modules, which contain thematic units of teaching, made available throughout the four years of the course, and which seek a dynamic articulation between practice and theory. It also presents transversal issues, termed 'sap' by the lecturers. The saps surpass the modules and aim for the broadening of the students' knowledges^(^ 5 ^)^. Among these, there is the Methodology of Care, the topic in which this study was based.

The reflection on the training for the SAE was based in the principles of Complex Thinking of Edgar Morin. Considering this theme in this perspective entails considering the teaching of SAE as a soil of uncertainties, where the knowledge must be rooted, constantly watered, and be shown to be open to pruning, whenever this may be necessary. Through Complex Thinking, it is sought to understand in the teaching of SAE the capacity for contextualization among the elements which compose this knowledge^(^ 6 ^-^ 7 ^)^.

The term 'complex' used by Morin comes from the Latin *complexus*, and means what is woven together and has the connotation of web^(^ 6 ^)^. It does not refer to something which is not simple or complicated, but, rather, towards what is inseparable. The challenge of complexity is presented based in the need to: reconnect what is usually separated; contextualize the knowledge; and allow interaction between certainty and uncertainty. The complex needs to capture interrelations, distinct and conflicting realities, through a thinking which respects diversity, seeking to unite instead of simplify^(^ 7 ^)^.

In this perspective, the objective of this study was to analyze the perceptions of the students of an undergraduate course in nursing regarding their training in Systematization of Nursing Care. 

## Method

A comprehensive qualitative study was undertaken in an undergraduate course in Nursing, in a public state university in Brazil, which has used the integrated curriculum for 13 years. This design was selected as the comprehensive approach makes it possible to reveal the complexity of the contexts, according to the point of view of different actors and groups studied^(^ 8 ^)^.

The students from the four years of the course participated in the study. The inclusion criteria were: to be a student of the Nursing Course and to accept to participate in the focus groups^(^ 8 ^)^. Collection took place between the months of October 2012 and March 2013. 

In order to undertake the focus groups, an invitation was issued to the students in the classrooms, with presentation of the study's objective and interests by the principal researcher. After surveying those interested, a further invitation was made, by email and by telephone, informing them of the date and place of the activity. On the day of each focus group, besides the principal researcher, two observers and one lecturer supervising the study were present, who made annotations which served as support for the transcriptions of the groups. 

Only one focus group session was held per university year, due to the saturation of the empirical data, obtained in each session, totaling the participation of 32 students, with a mean of eight students per university year. The guiding question was: "Tell me what comes into your mind when you think about SAE". Based on the discussions generated, it was sought to deepen the following questions: What is the relationship of SAE with the work of the nurse? What had they learned about SAE, until the present time, on the course? How did they evaluate the teaching of SAE in the integrated curriculum? Suggestions for promoting the teaching of SAE. Finally, the researcher returned to the principal content discussed in the group, so as to allow reformulations or additions by the students. 

The group discussions were recorded and filmed. The filming was used as a support resource for the elaboration of the transcription of the empirical material. The mean time for undertaking each group was one hour. The discussions held in the focus groups were transcribed in full and submitted to Bardin's thematic content analysis^(^ 9 ^)^. During the analysis, in the participants' discourses, themes which had to do with the learning of SAE were sought, so as to construct a sequence of these over the university years. Following that, the themes identified were grouped in three categories, and discussed in accordance with the theoretical framework adopted in this study: the principles of Edgar Morin's Complex Thinking - hologramatic, recursive, and dialogic^(^ 6 ^)^.

The study was approved by the Research Ethics Committee of the State University of Londrina, Paraná, Brazil, under Opinion 84180/2012, in accordance with CAAE 06270612.2.0000.5231. All the participants signed the terms of consent. The accounts were identified by the letter E, related to the undergraduate student, followed by the numbers corresponding to the university year and to the participants in the group. 

## Results and Discussion

The study participants were: 8 students from the first university year, 6 from the second, 7 from the third, and 11 from the fourth; ages varied between 18 and 26 years old, and 7 students were male, and 25 female. Of the total, only two mentioned having a professional activity, while the other students dedicated themselves purely to their studies. 

The analysis of the transcriptions of the focus groups made it possible to construct the categories: the activities of teaching and learning of SAE: a hologramatic reading; the student's view regarding her training in SAE: a recursive reading; and the student's feelings regarding learning SAE: a dialogic reading. The categories presented the perceptions of the students regarding their training in SAE, both in the years in which they were studying, and in those in which they had already studied. 

### The activities of teaching and learning of SAE: a hologramatic reading

The students of the first and second university years had difficulties in answering regarding what they had learned about SAE up to that time, on the course. Thus, it was necessary to explore what the activities were which these were undertaking or had been undertaking until that moment on the course. When they mentioned the medical history and the physical examination, the researcher deepened the discussion, trying to identify whether the interviewees related these activities to SAE; however, the students were not able to relate these. 

In relation to the physical examination, the students of the first university year reported having participated in classes in which the group was divided in order to learn about the physical examination of each system in the human body, in different stations, laid out in a laboratory. Following that, they undertook the practice of taking the medical history and of the physical examination of a system, in units of a teaching hospital, in pairs and under the supervision of a lecturer. *They gave us two weeks to learn the theory of the physical examination, and one day to go to the hospital to undertake the practice (E1-1).*

The students of the second university year stated that, in the first year, they had had theoretical classes on the physical examination and the presentation of seminars. Even in the second university year, they were not able to relate these activities to SAE and to the practice of the nurse. *In the first year, we presented a seminar, the group had to read the book on SAE and make a slideshow to present (E2-1).*

Regarding the second university year, they reported that they had undertaken the medical history, the physical examination and some nursing techniques; however, they still had difficulties relating these to SAE. The practical classes took place in a teaching hospital and in primary healthcare centers. *In the primary healthcare center, we undertook guidance, we measured blood pressure, we gave advice, we undertook physical examinations. With diabetic patients, we sought to see their feet, to see whether everything was okay, that there weren't any injuries (E2-3).*

The students of the third university year stated that, in the first university year, they had had theoretical classes on the physical examination and that they practiced on each other, and that in the second university year, practice was undertaken in placement fields. *We didn't have a close relationship with the hospital, so the physical examination was a class in which she brought slides and spoke, and this is something which if I don't go there, and touch the patient and the stethoscope, I am never going to learn (E3-5).*

For the students of the fourth year, the insertion of SAE had taken place in the second year, through a theoretical class and through undertaking nursing techniques. *The learning of SAE as well - in the first and second years, it isn't there, there are occasional classes (E4-2).*

It is observed that, in spite of the attempts to introduce - in the initial years of the course - elements which make up SAE, the students are not able to understand them as such, which reveals a lack of explanation regarding the relationship between the medical history and the physical examination and SAE. This difficulty is also reflected in the ability to grasp the issue. The fragmentation of the teaching brings with it the risk of not achieving the point of unity, that is, when the understanding of the whole is obtained. 

The hologramatic principal of the theory of complexity has as its model the hologram, according to which, one part of the image contains the whole of the object^(^ 6 ^)^. This principle transmits the idea that it is not possible to know the whole without knowing the parts, neither to know the parts without knowing the whole, as knowledge, in order to be pertinent, must be situated in a context^(^ 7 ^)^. The complexity proposes a change in the teaching, contrasting with simplifying and mutilating thinking, in search of pertinent teaching which contextualizes and aims for a broad understanding of the object^(^ 7 ^)^.

It has to be taken into consideration that there is relevance in the teaching in parts, when there is the objective of undertaking successive approximations for the development of skills in sequence. However, when one works based in this proposal, it falls to the lecturer to present to the students the pedagogical intention behind these activities, seeking to refer to the whole.

In relation to the third year of the course, the students reported having undertaken the medical history, the complete physical examination, the nursing diagnosis of the North American Nursing Diagnosis Association (NANDA), interventions from the Nursing Interventions Classification (NIC), implementation of care measures through nursing techniques and the nursing evolution, and recognize these elements as ways of undertaking the SAE. *In the third year we studied diagnosis, intervention, the nursing evolution and the reasons why one undertakes the physical examination (E3-2).*

The group also mentioned the insertion of the medical history and of the physical examination in primary care activities; however, still without relating these to SAE. After the discussions in the focus group, the students recognized SAE linked to childrearing, to consultations for prevention of cervical/breast cancer, and to prenatal care. *In childrearing, we examined the child, and afterwards would make a little progression report about her. And we did an SAE, whether we wanted to or not, it's just that we didn't have any notion that it was SAE (E3-3).*

I have noticed, I've just found out, that what we were doing was SAE, I had never made the link (E3-6).

The students of the fourth year stated that, in the third year, they had undertaken SAE associated with nursing diagnoses, prescription, and the nursing evolution. *In the third year you begin. You say hello to NANDA (E3-2).*

Even in the third year, when the students were already familiar with the expression SAE, and used it through the NP, the difficulty in seeing it in the context of primary care remained, a fact also mentioned by the students of the fourth year. This result reveals the difficulties which these have in distinguishing the content as a whole. This condition evidences the need of the lecturer to act as mediator between the new content and the students' prior knowledge. The fact that SAE is considered as a 'sap' reinforces the need for pedagogical intentionality in addressing it. 

It is noted that the different activities experienced by the students, in the primary healthcare centers, encompassed the undertaking of SAE; however, it is necessary that these should be conducted and guided effectively by the lecturers. Thus, meaning is given to the students' experiences, broadening understanding of the issue and allowing meaningful learning. The search for meaningful learning allows a real understanding of the issue, which will result in a deeper and more stable knowledge on the part of the students^(^ 10 ^)^.

The construction of the knowledge does not occur through a sum of knowledge, but through the transformation and organization of what has been learned. Complexity requires the search for unity, and often leads the human being to try to equalize the parts, erasing their specific characteristics. However, the characteristics of each part must be preserved such that, in this way, there may be full understanding of the whole^(^ 7 ^)^.

The evidence is that, during the undergraduate course, it is necessary to pay attention to how the content is addressed, such that successive approximations may not be seen purely as an agglomeration of new issues. In this way, when the stages of NP are undertaken, besides deepening the knowledge and the pre-existing practice regarding this knowledge, one must constantly relate these to SAE, that is, to the whole. 

In the fourth year, the students mentioned the nursing prescription, the nursing evolution and physical examination as means of undertaking SAE. These were undertaken in hospital units and primary healthcare centers. Even in the last year of the course, they were still developing skills for undertaking SAE. *I only learned to prescribe activities in the fourth year (E 4-1).*

It was observed that the issue of SAE is addressed ascendingly, as, in each university year, prior knowledge is returned to with presentation of new knowledge. However, it is fundamental for the students to perceive the relationship existing between the knowledges, the skills developed, and SAE, given that the implementation of this last required skills which help the nurse to identify signs which can support her actions, which qualifies the management of the care^(^ 11 ^-^ 12 ^)^.

One path for mitigating this lack of relating is in the understanding that, over the university years, the new knowledge on SAE must be contextualized if the students are to manage to make this content relevant.

These suggestions also extends to the other schools of nursing. Although many schools also present traditional curriculums organized by courses, it is fundamental that these should cover the importance of training for use of SAE in professional practice. It is in this context that this study tries to encourage the nursing courses to accept responsibility for improving the teaching of SAE.

### The student's view regarding her training in SAE: a recursive reading 

During the undertaking of the focus groups, the students made various points, evaluations and suggestions regarding their training in SAE. It is evident that listening to the student is one way of assisting the lecturers and course coordinators to evaluate the efficiency of the teaching process and, consequently, to propose improvements for the organization of the same. 

In the first year, the students' evaluations made clear the need for a greater number of practical activities in the laboratory, and more time for studying, prior to activities in placement fields. They requested greater preparation for the first physical examination, as this is striking. *It wasn't because of lack of dedication on our part, or on the professor's, sometimes it also involves time, so they shouldn't just speak about theory, they should really say how it is to be facing a patient, and what the fears are which may arise (E1-7).*

The students of the second year also presented suggestions for the first year. They mentioned difficulties in the theoretical classes, through the method of seminars, as these presented the themes based in research on the Internet, without any in-depth understanding of the issue. The classes took place at the time in which they were not able to relate the physical examination to the other classes which they were having. *In the seminar on the physical examination, we knew because we read the slide, in practice we had no notion of what we were talking about because we had never done it, so it was totally wrong, this issue of giving the physical examination last year, and the practice this year, because we had forgotten a lot of stuff. I think that they should have given the practice and the theory together, to make it easier (E2-2).*

In relation to the second year, the students stated that they had had difficulty in making annotations in the hospital records, due to not knowing how to identify the relevant aspects of an annotation, and evaluated that the theoretical class on the physical examination is extensive and has too much content. Furthermore, they mentioned that it is necessary to contextualize why the activities are undertaken, in a more meaningful way, and to improve the relationship between theory and practice. These points were also mentioned by the students of the third year. *We spoke to the coordinator of the seminar module, about the lack of practice. She saw that we didn't have the knowledge to undertake the physical examination (E2-4).*

In expressing their opinion on the characteristics which could improve the teaching of SAE, the students refer to an action which could result in changes in the organization of the course. In this way, cause and effect, that is, the teaching and the learning, abandoned the linear dimension and achieve a retroactive position, in which the learning influences the teaching^(^ 7 ^)^.

The recursive principle is explained as a process in which product and effect are, at the same time, cause and producer of what produces them^(^ 6 ^)^. In this way, the view of the student and of the lecturer as beings which exercise their roles in isolation, the lecturer being the retainer of knowledge, who dispenses the knowledge, and the student, a passive personality in the process of teaching, is transformed. There is no longer professor/teaching, on one side of the process, and the students/learning, on the other; both are inseparable and interdependent, as one influences the actions of the other. 

Recursivity, in the teaching of SAE, in the first years of the undergraduate course, confirms something mentioned above, that is, the needs to situate this knowledge in a context which covers the whole. In order to infer whether the teaching is meeting the students' needs or not, frequent times for evaluation and listening to the same must be incorporated. Student-centered learning, the articulation of theory and practice, and formative evaluation which respects the individuality of the student, constitute challenges to the context studied, and also to other Brazilian nursing schools^(^ 13 ^)^.

It was observed that on many occasions the students complained of the activities for which they were responsible, strongly requesting more theoretical classes and more study time. The analysis of these situations makes it possible to reflect that the lecturer must support and facilitate the students' learning, but not take from them the active search for the learning itself. However, the lecturer must not distance herself to the extent that the students feel insecure and alone. 

The third-year students mentioned not remembering SAE in the previous years. They complained of the few theoretical classes throughout the course, and that the same were decontextualized from the other content studied. As suggestions, they proposed a better division of the topics of the classes. *The theory was weak, it was two heavy classes, it was thrown together (E 3-6).*

In addition to this, they mentioned that it is necessary: for there to be a standard for evaluating the nursing evolution, on the part of different lecturers; of undertaking these with a greater number of patients; and the making available of longer periods for the practice of the nursing evolution. *Each professor had their own way of doing the diagnosis and the nursing evolution (E3-6).*

The fourth-year students reported having difficulties in undertaking the nursing evolution and that the insertion of this at the end of the course hindered their understanding. They considered the role of the lecturer to be important; however, they wished for greater contact with the evaluation of critically-ill patients. They also evaluated that this is presented in a fragmented way in the course. At the end of the year, they reported difficulties in undertaking the nursing prescription and the nursing evolution. *We did the nursing evolution as a nursing annotation, the nursing evolution over 24 hours, we didn't do this because we didn't know that we had to. There was just one class, which is not enough to have a notion of how you do it all, even if the class is good (E 4-3).*

In relation to the teaching of SAE in the other years, various suggestions were made, including: using case studies for greater retention, and for them to be presented to SAE in a clearer way, from the first year onward. In relation to the second year, they suggested better contextualization of the theory class. For the third year, they requested: earlier contact with nurses working in the field and with their activities; undertaking of prescription of care, rather than just implementation; undertaking of more nursing evolutions instead of annotations; and greater standardization on the part of the lecturers in the evaluation of the nursing evolutions, so as to preserve the academic style. 

In the third and fourth years, the students return to simplifying thinking, when SAE is divided into parts, which are often isolated from the context, over the course. Complex thinking, in contrast with the mutilation which simplifying thinking causes, goes through a path of maximum integration^(^ 6 ^)^. The teaching of SAE, fragmented over the years, was perceived by the students as a bundle of unconnected fragments, as the understanding of what the stages of the nursing process, in the context of SAE, and their relationship with the nurse, takes half the course. 

The students of the fourth year confirmed that they still saw SAE as an activity practiced only by the students, as they did not witness it being undertaken by the nurses in the field. The situation is also found in the literature, which presents various rationales, indicated by the professionals, for the non-implementation of SAE, including: the reduced number of professionals; the overload of work; the high number of patients; the inappropriate conditions of the work; and the bureaucracy^(^ 14 ^)^.

In one American study, researchers identified that 75% of the nurses interviewed said that their practice was mainly based in personal experience of nursing, rather than in the literature or nursing research. The lack of time is presented as one of the difficulties for change in the work of the interviewees^(^ 15 ^)^. This finding reconfirms the nurses' difficulty in seeking evidence-based practice, and the obstacles which permeate the undertaking of SAE by the professionals. 

Many activities are performed by the nurses in their professional practice, but it is understood that the task of constantly seeking to develop SAE, responsibly and in a dedicated manner, so as to help the students to perceive the need for its implementation and consequently for them to use this highly important tool in their professional life, falls to the schools of nursing. The implementation of SAE can afford quality care, which is reflected in the care for the patient and for the community, in their health-illness process^(^ 12 ^)^.

In summary, in this category, it was perceived that teaching and learning are constituents of a process. In this, the interactions produced result in its transformation. The perception of recursivity, in the teaching of SAE, depends on an openness to new ideas and changes; otherwise, the results would entail a mere refusal of the problems raised^(^ 7 ^)^.

### The student's feelings regarding learning SAE: a dialogic reading 

The construction of knowledge on SAE is permeated by various ambiguous feelings, which interfere in putting it into effect. The dialogic is the third principle presented by complex thinking, and this consists of uniting ideas which are apparently contrary, that is to say, the teaching needs to relink concepts which are separated due to their ambiguity^(^ 7 ^)^.

The author of complex thinking exemplifies this idea based in the notion of order and chaos. Simplifying thinking would separate two antagonistic concepts, in search of order, as a solution for the chaos. For complexity, these terms are complementary and inseparable, as knowledge needs to coexist with uncertainties^(^ 6 ^)^.

In the first year, the feelings described by the students were: insecurity, unpreparedness, tiredness and - in spite of the satisfaction due to being close to the nurse's activity - being uncomfortable approaching the patient for the first time. *Because we felt very insecure [...] We had no notion of how it would be, being in front of the patient [...] Because it was the actual first time you know, and to be dealing with another human being is not easy (E1-2).*

In the second year, feelings of unease, uncomfortableness and unpreparedness continue, mainly in undertaking the physical examination. *On the day when I went to do the physical examination, in the beginning I was kind of, not ashamed, but it's complicated, because I didn't know (E2-5).*

In the third year, the feelings of insecurity remain for some students, while for others, however, stress appears, due to the doubts which emerge in undertaking SAE. *I felt a lot of difficulty, because they requested a far more detailed physical examination, so much so that I had to go and study it myself (E3-5).*

In the fourth year, the predominant feeling was insecurity in undertaking SAE. *We don't know how to do SAE, we don't know, we learn it in practice (E4-8).*

The students, over the four years, faced conflicting feelings in relation to the construction of their knowledge. Unpreparedness and insecurity permeated the SAE practices, and ignoring or belittling these feelings entails suffering. 

Complexity explains order as something constant and repetitive, while chaos is seen as something unpredictable and irregular. A world of order would lack renewal and change and would, therefore, not shelter the human being^(^ 6 ^)^. For this reason, teaching must seek to understand chaos to be able to organize. 

The feelings expressed by the students are, as it were, a path for rethinking the teaching of SAE over the years of the course. The lecturers must make use of these uncertainties and insecurity to explore the issue, making the discussions more meaningful and giving new meaning to the undertaking of SAE. 

The students reported a time of dialogue regarding the feelings experienced in the learning of SAE in the first year. This result reveals the need to reserve more space for this type of action in the modules throughout the course. 

The act of listening goes far beyond simple hearing, as it means grasping the meaning of what is said. Listening, often, is hindered by the common habit of comparing life experiences, values and culture with the history presented by the other^(^ 16 ^)^. The field of education is no different from that of other situations where listening is necessary. The lecturers, besides fostering the search for knowledge, also need to provide moments of listening, with a view to supporting the distress which appears in the learning process. 

It is understood that, in linear teaching, the content is presented and a receiver must develop skills for putting them into practice. In this context, feelings and uncertainties may be suppressed. This posture does not encompass the real needs of the human being, who, once inserted in a process, participates in it with her ideas, emotions and traditions. Thus, it is necessary for the understanding of the process to be considered, so that it may become possible to transform what is not clear and, in this way, to achieve improvements in the teaching and learning. 

## Conclusion

The students' perception regarding SAE, over the duration of the course, is geared towards the learning and towards the practice of the NP and of the nursing consultation undertaken since the first year. It was possible to observe that the content was presented in a fragmented way, causing some conflicts in the students, who only understood the reason for this knowledge from the second half of the course onward. In order to resolve this difficulty, the contextualization of SAE and of the elements which allow it to be put into effect is of fundamental importance, as it will allow a more pertinent learning.

The evaluations indicated by the students call attention to new aspects to be taken into consideration in the organization of the content regarding SAE which, in spite of permeating all the years of the course, still need improvements. The intersections found may be smoothed out through a reorganization of the content and the relationship of these with the SAE, through the mediating action of the lecturer, thus achieving greater meaning for the students.

Coping with the paradoxical feelings which are presented in the process of appropriating knowledge of SAE, during training, is also important, as this will allow new interpretations by the students, which will broaden their view on the study object. The lecturers must incorporate moments of listening and dialogue, over the course of the modules, so as to guide the students in coping with the difficulties found. 

The training in SAE, in the perspective of complex thinking, allows a more accurate view, which can allow more effective and critical teaching, applicable to other schools of nursing. To improve the development of this issue, without reducing it to concepts, is a challenge which will culminate in the broadening of the understanding and of the practice of this fundamental tool of work of the nurse. 
